# Processable dodecylbenzene sulfonic acid (DBSA) doped poly(N-vinyl carbazole)-poly(pyrrole) for optoelectronic applications

**DOI:** 10.1080/15685551.2016.1271086

**Published:** 2016-12-27

**Authors:** W. A. Hammed, M. S. Rahman, H. N. M. E. Mahmud, R. Yahya, K. Sulaiman

**Affiliations:** ^a^ Faculty of Science, Department of Chemistry, University of Malaya, Kuala Lumpur, Malaysia; ^b^ Faculty of Science, Department of Physics, University of Malaya, Kuala Lumpur, Malaysia

**Keywords:** Chemical polymerization, poly N-vinyl carbazole, dodecyl benzene sulfonic acid, polypyrrole, biphasic, ammonium persulfate

## Abstract

A soluble poly (n-vinyl carbazole)–polypyrrole (PNVC–Ppy) copolymer was prepared through oxidative chemical polymerization wherein dodecyl benzene sulfonic acid (DBSA) was used as a dopant to facilitate polymer-organic solvent interaction and ammonium persulfate (APS) was used as an oxidant. Compared with undoped PNVC–Ppy, the DBSA-doped PNVC–Ppy copolymer showed higher solubility in some selected organic solvents. The composition and structural characteristics of the DBSA-doped PNVC–Ppy were determined by Fourier transform infrared, ultraviolet–visible, and X-ray diffraction spectroscopic methods. Field emission scanning electron microscopic method was employed to observe the morphology of the DBSA-doped PNVC–Ppy copolymer. The electrical conductivity of the DBSA-doped PNVC–Ppy copolymer was measured at room temperature. The conductivity increased with increasing concentration of APS oxidant, and the highest conductivity was recorded at 0.004 mol/dm^3^ APS at a polymerization temperature of −5 °C. The increased conductivity can be explained by the extended half-life of pyrrole free radical at a lower temperature and a gradual increase in chain length over a prolonged time due to the slow addition of APS. Furthermore, the obtained soluble copolymer exhibits unique optical and thermal properties different from those of PNVC and Ppy.

## 1. Introduction

In recent years, vast research interests have led towards preparing new composites based on conducting polymers to harness their excellent properties and simple synthetic routes. Conducting polymers are useful in optoelectronics, sensors, as well as chemical displays, such as biosensors, photodiodes, organic light‐emitting diodes, field‐effect transistors, supercapacitors, and photovoltaic cells [1–6]. In a *π*-conjugated polymer, such as polypyrrole, polythiophene or polyaniline, alternating single and double bonds of the polymer backbone control both the transfer of electron and the energy levels. Also, the delocalized *π*-bonds electrons in conducting polymer backbones are responsible for strong interchain charge transfer interactions, by characterizing their exceptional combination of electrical and mechanical properties. However, poor processability from organic solvents, environmental instability, and poor mechanical properties are among the limitations to potential applications of conducting polymers.

Furthermore, previous reports have shown that copolymerization of pyrrole (Py) with n-vinyl carbazole (NVC) yielded copolymer with improved thermal stability, conductivity, electrophotography [7], and dielectric properties. In the case of poly(N-vinyl carbazole) (PNVC), the justifications were based on its good photoconductivity [8,9] and thermal stability (up to 300 °C), albeit it possesses a wide band gap and poor electrical conductivity (10^−10^–10^−16^ S/cm^−1^). PNVC is a hole conductor, which has also demonstrated electron mobility upon doping with iodine [10]. In fact, studies have suggested that PNVC possesses the ability for effective hole and/or electron mobility. Nevertheless, a major drawback to these properties is the extreme brittleness, which however, has prompted many efforts towards improving both the mechanical and the chemical processing of PNVC. Many research efforts have focused on the development of copolymers, composites or blends containing PNVC moiety in order to harness its hole transport and thermal stability properties. Such materials have been developed via chemical or electrochemical polymerization of NVC monomer with inorganic and high electrical conducting materials, such as graphene [11], fullerene [12] or carbon nanotubes [13] to achieve composite material with excellent luminous and conducting properties. On the other hand, due to its solubility in common organic solvents like chloroform, benzene, tetrahydrofuran, and toluene, researchers have considered copolymerizing PNVC with conducting polymers that are inherently intractable, perhaps, the presence of PNVC can induce solubility of the resulting copolymer. In 1997, Narayan and Murthy studied the absorption, the emission, and the short circuit current of bilayer device based on PNVC-P3HT. The device displayed additional PL features with respect to PNVC single layer device. In addition, recently, Alimi et al., synthesized copolymers based on PNVC and polymers, such as poly(3-methyl thiophene) [11], poly(3-hexyl thiophene) [12], and poly(p-phenylenevinylene) [13]. All the copolymers products are solution processable with PNVC-P3HT and PNVC-PPV exhibited optical and electrical properties suitable for optoelectronic applications. The earliest work on copolymer and composite based on NVC and Py was reported by Toppare et al. [14] PNVC–Ppy copolymer and composite were prepared via electrochemical polymerization. They reported that the conductivity of the products was within 10^−1^ and 10^−3^ S/cm without mentioning the solubility of their products. Later, Biswas and Roy [15–18] conducted exclusive findings on the properties of PNVC–Ppy by using different oxidants and in varying the reaction medium, although the copolymer showed improvement in thermal stability, as well as morphological and conductivity properties; but none of the polymer products was soluble in organic solvent. On top of that, further attempt made by Biswas and Ballav to copolymerize NVC and TP also resulted to intractable polymer nanocomposite, but with improved thermal properties in relation to the homopolymers [19]. Besides, Wan et al., synthesized a flexible film of PNVC–Ppy with room temperature electrical conductivity as high as 10 S/cm by using laser-electrochemical polymerization. Yet, the authors did not mention any report about the solubility of the resulting polymer [20].

Polypyrrole (Ppy) has been given wide focus and its commercial application is expanding by the day; owing to its excellent electrical conductivity [21,22] and superior environmental stability [21]. However, polypyrrole is obtained as an intractable powder that is difficult to process in solution. Conjugated backbone, the condition for its metallic conductivity, is also responsible for its insolubility. Thus in order to obtain soluble polypyrrole product while maintaining its electrical conductivity, non-substituted pyrrole monomers are replaced with alkyl substituted monomers, which are proposed to reduce the interchain interaction between the chains of polypyrrole molecules in doped state [22,23]. Unfortunately, the presence of long alkyl chain on the pyrrole causes a stearic hindrance that affects the planarity of the resulting polymer. As a result, *π*-orbital overlaps are greatly affected and they are less available; causing a significant reduction in the polymer conductivity. The soluble polypyrroles obtained, nonetheless, exhibited low electrical conductivity.

However, limitations to commercial applications of conducting polymers, especially polypyrrole and polyaniline, have recently been reduced by successful synthesis of conducting polymers that are soluble in common organic solvents, such as m-cresol, chloroform, and dimethyl formamide (DMF). Moreover, surfactant anions were used as counter ions that induced their solubilities [24]. At present, dodecylbenzenesulfonic acid (DBSA) and camphorsulfonic acid are used as dopants to obtain free-standing polyaniline film from emeraldine base solution [25]. In addition, soluble polypyrrole have been synthesized via one-step chemical oxidative polymerization [26]. Incorporation of surfactant anions into the backbone of polypyrrole allows the long alkyl chain of the dopant anions to serve as a spacer that expands the polymer chain to enable diffusion of organic solvents into the spaces created by the dopant molecule [27]. In optoelectronics, especially organic solar cell fabrication, conjugated polymers are often used as the donor (or acceptor) materials in the device active layer. In fact, properties, such as good redox potential, high electrical conductivity, environmental stability, and solution processability, which are also characteristics of doped polypyrrole, can be harnessed if doped polypyrrole is used in the active layer of organic solar cell.

Dopant stabilized polypyrrole are usually prepared in water by using ammonium persulfate (APS) oxidant. Hence, we hypothesized that if the polymerization is carried out in the presence of NVC, solution processable copolymers soluble in common organic solvents could be yielded. This could widen the potential applications of pyrrole/carbazole derivatives, especially in optoelectronics. NVC is insoluble in water, but soluble in various organic solvents, including acetonitrile, which is miscible with water. Therefore, in order to facilitate an interaction between NVC and APS oxidant, polymerization medium could be a mixture of water and acetonitrile. In this regard, this paper presents the chemical synthesis of soluble PNVC–Ppy copolymer in a doped state. The copolymer was synthesized in a biphasic system that consisted of both water and acetonitrile with a controlled amount of APS oxidant to initiate the polymerization process and DBSA, attached to pyrrole molecule, to induce solubility. As a result, the copolymer displays solubility in dimethyl sulfoxide (DMSO), DMF, and chloroform. In addition, the new copolymer exhibits other properties for application as an active layer in optoelectronic devices. Data obtained from X-ray diffraction analysis (XRD), FTIR spectroscopy, FESEM, and TGA are presented to examine its structural, morphological, and thermal properties.

## 2. Materials and methods

### 2.1. Materials

Pyrrole (Merck) was freshly distilled and stored in dark cool condition. N-vinyl carbazole (Aldrich Chemistry), APS (Acros Organics), and dodecyl benzene sulfonic acid (DBSA, Acros Organics) were used as received. All the solvents were of analytical grade.

### 2.2. Preparation of DBSA-doped PNVC–Ppy copolymer

Polymerization of PNVC–Ppy copolymer was carried out at chosen temperatures by using APS as the oxidant. 0.01 mol DBSA was dissolved in 80 ml of acetonitrile. To the above solution, 0.02 mol of NVC and 0.02 mol of pyrrole dissolved in 80 ml of acetonitrile were added. The mixture was stirred vigorously for 5 min and then kept at 0 °C. 0.004 mol of APS in 40 ml ACN/water (1:1) mixture was added dropwise. The gradual addition of the oxidant turned the monomer solution to a dark-green color, indicating the on-set of polymerization. The solution, later, turned dark-brown after 6 h of reaction. The chemical-oxidative polymerization of the copolymer proceeded for 6 h and was terminated upon the addition of methanol. The dark-brown polymer product was filtered and sequentially washed with 10% HCl, distilled water, acetone (to leach out excess DBSA), and toluene (to remove unreacted NVC monomer and PNVC homopolymer). The final product was filtered and dried in a vacuum oven at 60 °C.

### 2.3. Characterization

The polymerization yield was calculated based on the following formula:

$$\;\text{yield}\left(\%\right)=\left(\frac{m_{1}}{m_{2}}\right)\times 100\%$$

where *m*_1_ is the weight of PNVC–Ppy copolymer, and *m*_2_ is the weight of NVC + pyrrole monomers. The current–voltage measurements were performed by using a Jandel RM3000 Test Unit and the electrical conductivity was calculated by using the following equation:

$$\sigma =V^{-1}I\left(\frac{\ln2}{\pi \;d_{n}}\right)$$

where *V* is the applied potential measured in volt, *d*_*n*_ is the thickness of the pellet measured in cm, and *I* is the current in ampere.

Field-emission scanning electron microscopy (FE-SEM) images were taken to examine the morphology of the PNVC–Ppy by using Hitachi SU-8220 microscope, while FT-IR spectra were measured with Perkin Elmer FTIR-Spotlight 400 spectrometer. Optical density measurement was performed by using a Shimadzu UV-2600 spectrometer, in the range of 200–1100 nm. The XRD spectra were measured on a PANalytical EMPYREAN model diffractometer with Cu-K radiation. Meanwhile, thermogravimetry was performed on a Perkin Elmer TGA 6 model instrument from 25 to 900 °C at a heating rate of 10 °C/min under atmospheric condition, whereas electrical conductivity measurement was taken by using four-point probe method on the pellets (0.15 g, diameter 1 cm) of the homopolymers and the copolymers compressed (pressure, 15 bar) from their respective powdered samples. In this work, molar ratios APS/NVC–Py = 1 and DBSA/NVC–Py = 0.5 were used for all the characterizations. The reaction temperature and the time were −5 °C and 18 h, respectively. Solubility of the solvent was determined by dissolving 10 mg of the copolymer product in 20 ml of each of the selected solvents (DMOS, DMF, chloroform, THF, chlorobenzene) and then, ultrasonicated for 20 min. The solution was then filtered through a 1 μm Teflon membrane filter and the filtrate was transferred to a previously weighed glass plate where the solution was left to evaporate and solubility was determined.

## 3. Results and discussion

### 3.1. Roles of APS concentration on conductivity and yield

**Scheme 1.**
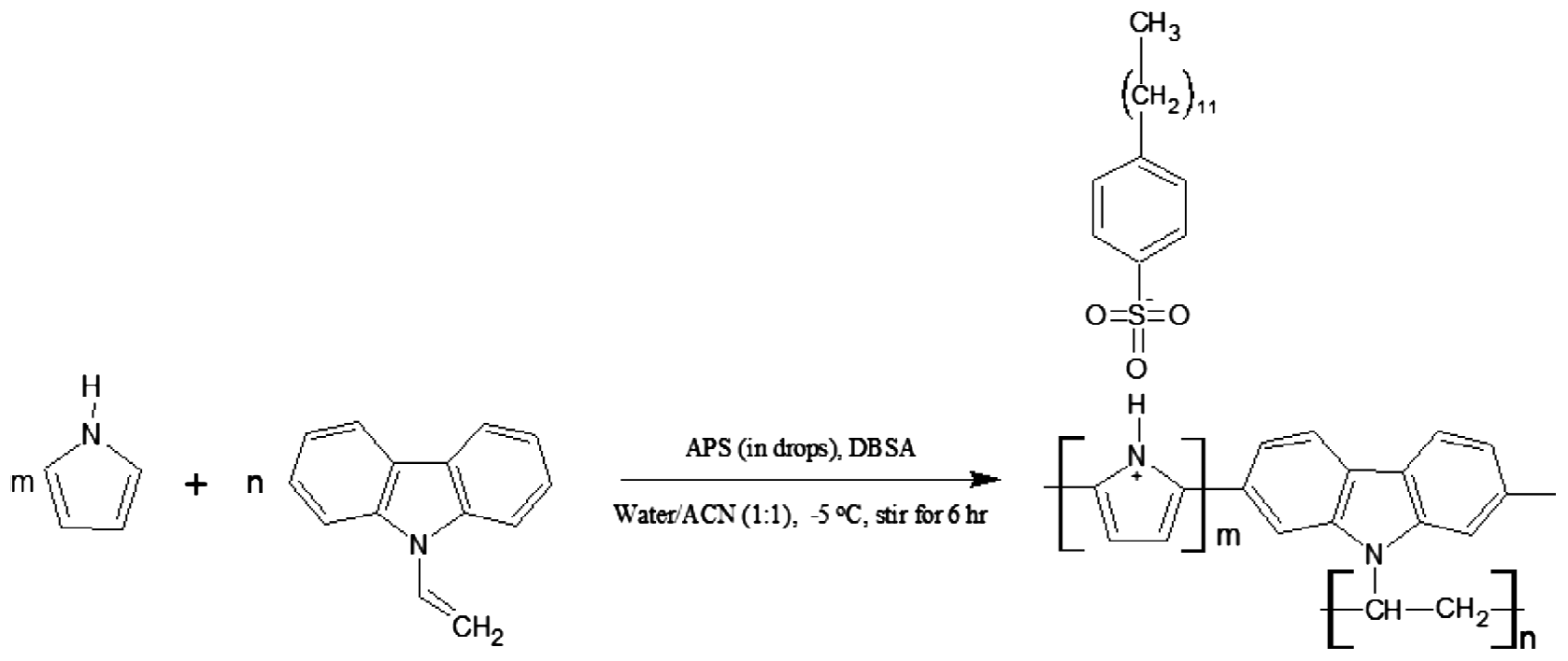
Synthesis of DBSA-doped PNVC*–*Ppy.

The synthesis of doped PNVC–Ppy copolymer is shown in Scheme 1. The polymerization medium was carefully chosen to facilitate an interaction between the monomers and APS oxidant. Interestingly, no precipitate was formed when APS was added to the mixture of water and acetonitrile. Moreover, the solubility of APS in water did not prevent the miscibility of water and acetonitrile It has been established that the yield and the conductivity of conducting polymers are affected by certain factors, such as monomer to oxidant ratio, duration, solvent, oxidant, and reaction temperature. For instance, P. A. Steven [28] obtained 100% yield of polypyrrole at optimum ratio (2.4) of iron(III) to pyrrole monomer. Also, polymerization at a short period of time yielded enhanced result when carried out at low temperature [29].

**Figure 1.**
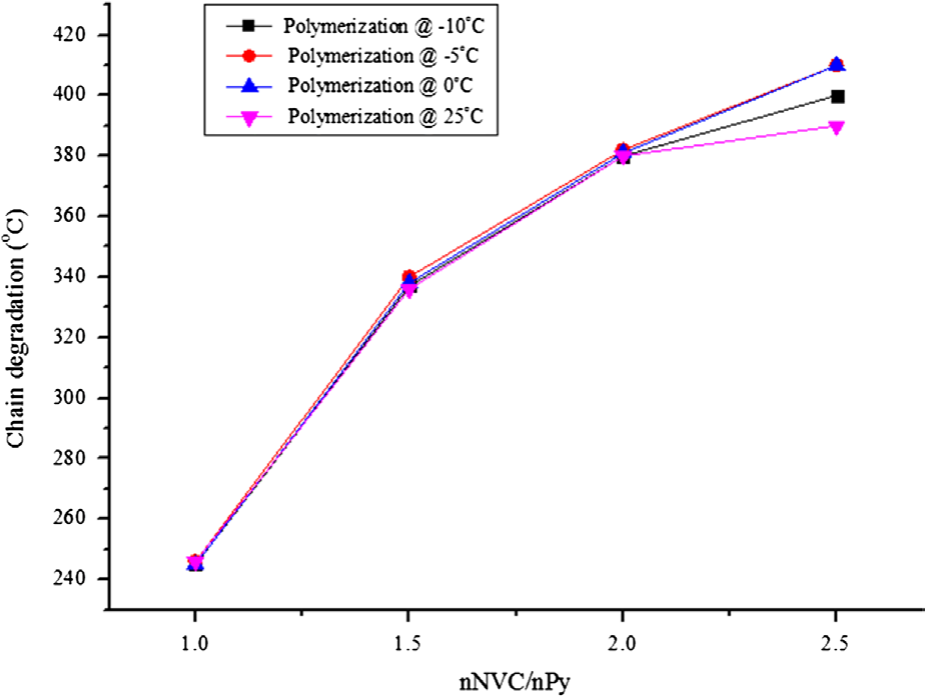
Thermal stability of PNVC–Ppy copolymer vs. NVC concentration at different temperature.

Additionally, in order to establish the effect of the APS concentration on the yield and the conductivity of DBSA-doped PNVC–Ppy, we first investigated the relationship between the amount of NVC and the thermal stability of the polymer product. As expected, there was a linear relationship between the weight ratio of NVC and the thermal degradation of copolymer chain (Figure 1). Besides, the copolymer with the highest NVC content was the most stable to heat, while the copolymer with the least content of NVC degraded faster.

**Figure 2.**
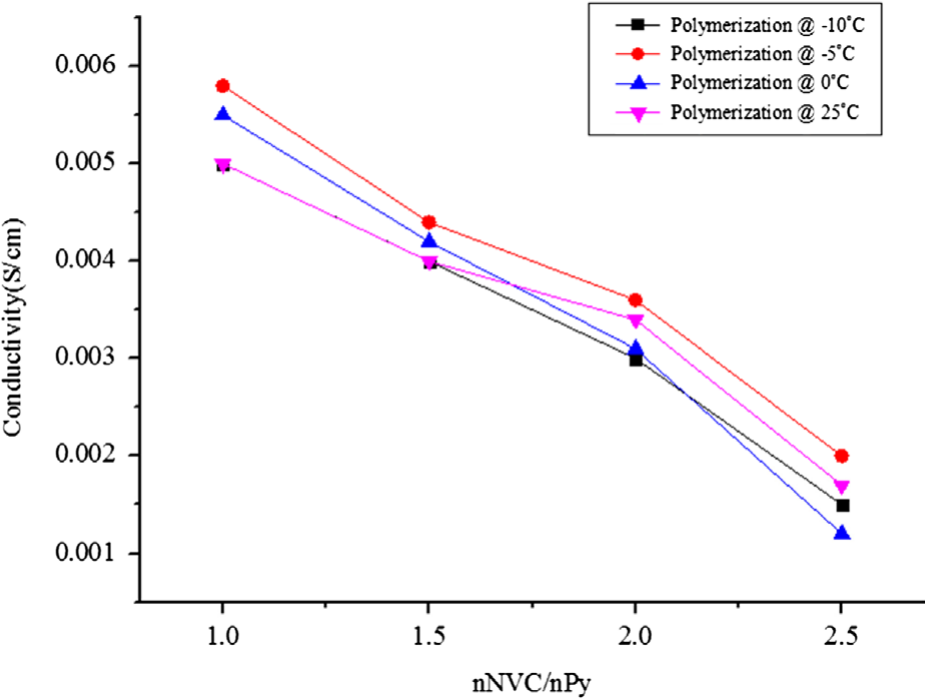
Electrical conductivity of PNVC–Ppy copolymer vs. NVC concentration at different temperature.

This result was further compared with electrical conductivity of the copolymer with varying concentrations of PNVC (Figure 2). The electrical conductivity of the copolymer decreased with increasing concentration of PNVC. However, the copolymer synthesized at mole ratio *n*_*NVC*_/*n*_*Py*_ = 1.0 had better thermal stability and moderate electrical conductivity at all the chosen temperatures. Hence, we investigated the role of APS concentration on electrical conductivity of PNVC–Ppy at a constant ratio of *n*_*NVC*_/*n*_*Py*_ = 1.0 and DBSA = 0.01 mol/dm^3^ while varying the APS concentration from 0.001 to 0.004 M at different temperatures.

**Figure 3.**
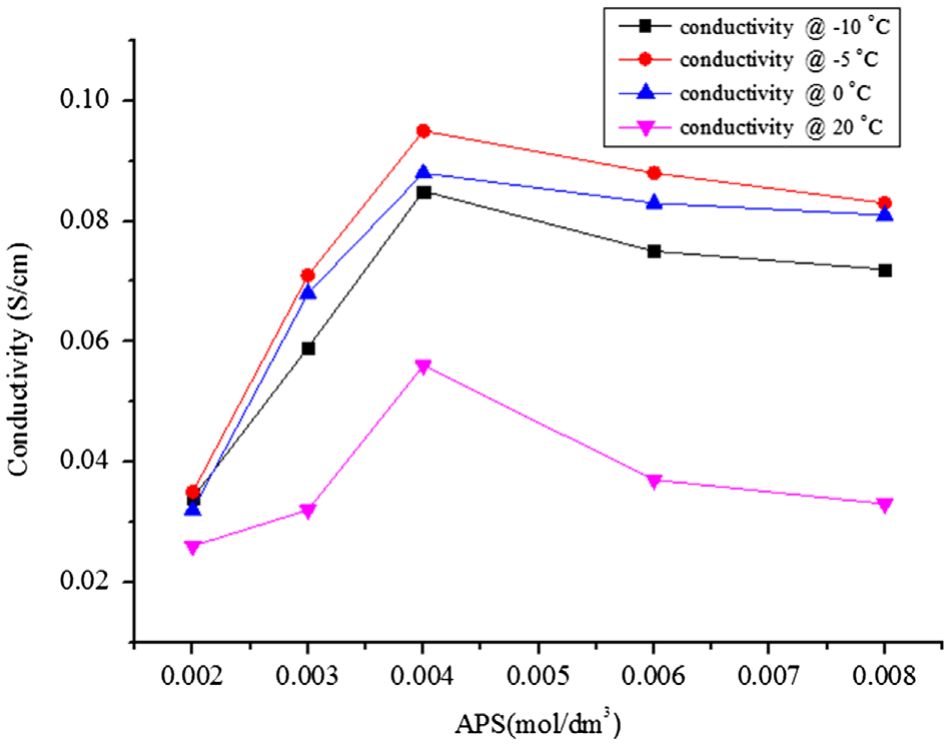
Electrical conductivity of DBSA-doped PNVC*–*Ppy vs. APS concentration at different temperatures.

Moreover, the electrical conductivity of PNVC–Ppy increased as the polymerization temperature decreased and with an increase in the concentration of APS oxidant (Figure 3). Polymerization carried out at −5 °C with an APS concentration of 0.004 mol/dm^3^ gave a better conductivity value, 0.095 S/cm. At temperature below the room temperature (−10, −5, and 0), the conductivity of PNVC–Ppy followed a similar trend. Slow addition of APS resulted in progressive chain growth; causing gradual increase in chain length over a prolonged period of time. Also, polymerization at a lower temperature extended the half-life of pyrrole free-radical cation or pyrrole oligomer for further random reaction either with each other or with carbazole unit. The conductivity, nonetheless, increased gradually until a maximum value of 9.5 × 10^−2^ S/cm was reached with 0.004 mol/dm^3^ APS at polymerization temperature of −5 °C and then, it started to decrease. However, since the polymerization was proceeded randomly, even at low temperature, high concentration of APS led to high oxidation of both pyrrole and NVC. On one hand, excessive oxidation of NVC could disrupt the pyrrole chain, thereby decreasing the conductivity of the copolymer. Also, pyrrole chain may be ruptured due to the high conversion rate to polypyrrole. Therefore, either or both of these conditions might lead to a decrease in conductivity of the copolymer while the concentration of APS increases.

**Figure 4.**
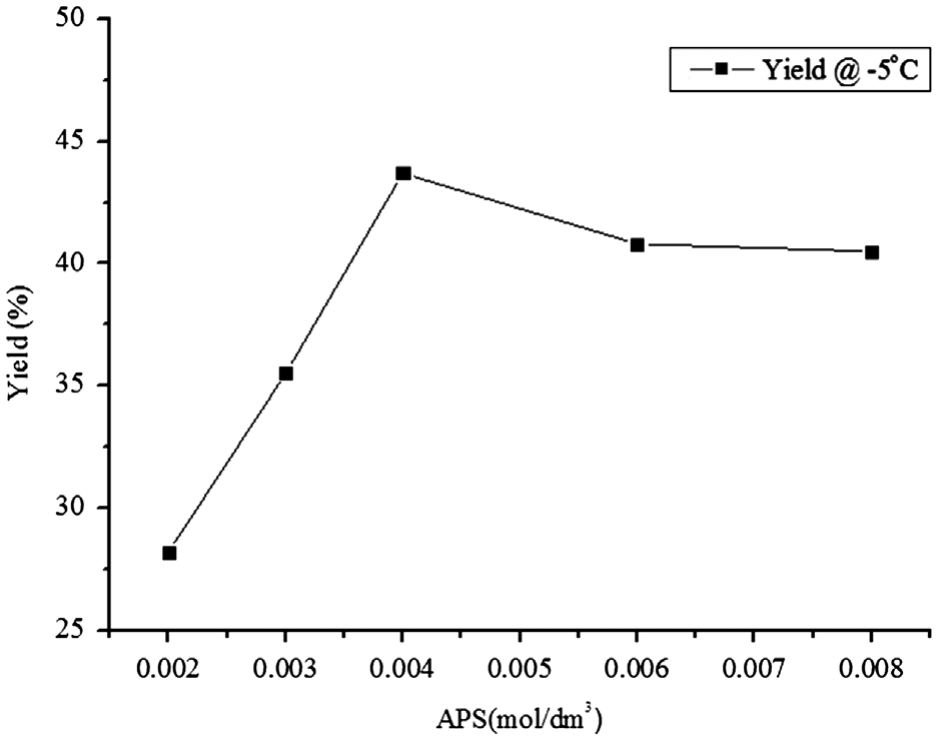
Yield of the copolymers vs. APS concentration at −5 °C.

The highest conductivity was recorded at a temperature of −5 °C. Thus, in order to obtain relevant information about the correlation between the oxidant concentration and the polymerization yield, the polymerization was carried out at temperature −5 °C at a fixed concentration of DBSA (0.01 mol/dm^3^) while changing the concentration of APS oxidant. The change in yield, with corresponding change in concentration of APS oxidant, followed a similar pattern to that of conductivity (Figure 4). The yield of PNVC–Ppy at the polymerization temperature of −5 °C first increased as the APS concentration changed from 0.002 to 0.004 mol/dm^3^. It then started to decrease at a higher APS concentration within the concentration range chosen in the present investigation.

The rapid increase in yield, thus, can be attributed to the rapid generation of radicals due to increased initiator concentration [30]. At concentration higher than 0.004 mol/dm^3^, lower yield was obtained, perhaps because the equivalent amount of effective APS needed for polymerization had already reached 0.004 mol/dm^3^ and there was insignificant contribution from the extra concentration of APS.

### 3.2. Roles of concentration of DBSA on solubility and conductivity of DBSA-doped PNVC–Ppy

**Figure 5.**
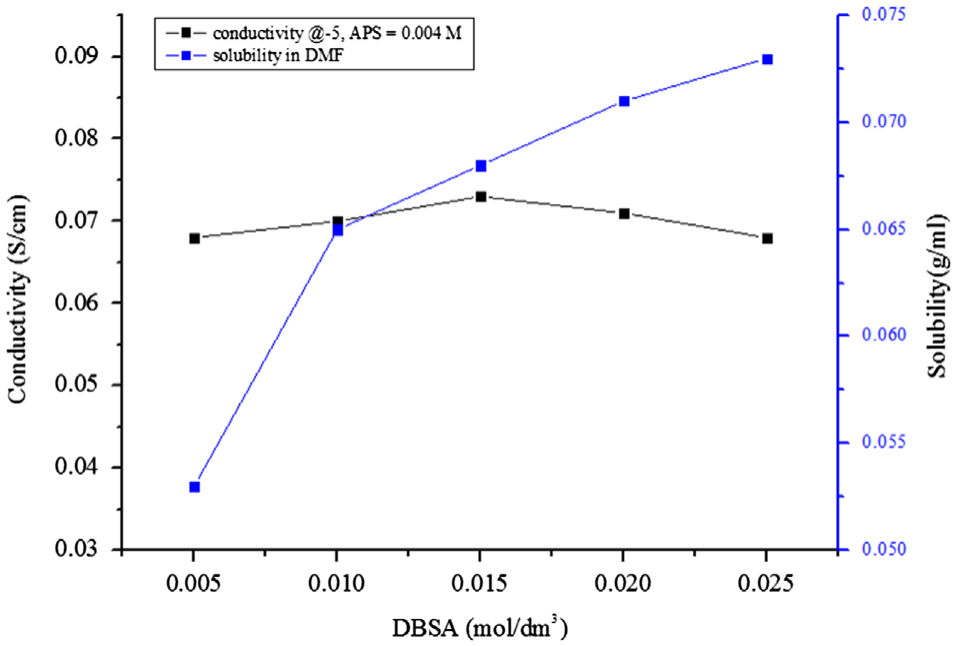
Solubility and electrical conductivity of DBSA-doped PNVC*–*Ppy synthesized with different DBSA concentrations.

DBSA was used as a dopant to stabilize the copolymer in organic solvents in order to obtain a soluble form of DBSA-doped PNVC–Ppy. While the concentration of persulfate oxidant was kept at 0.004 mol/dm^3^ and the temperature at −5 °C, the concentration of DBSA, with respect to conductivity and solubility of the polymer, was varied to obtain the optimum amount of DBSA required as a dopant. Moreover, since the copolymer dissolved better in DMSO, it was used as a solvent for this investigation. Conductivity of the copolymer showed little increase as DBSA concentration increased from 0.005 to 0.015 mol/dm^3^ (Figure 5).

At DBSA concentration higher than 0.015 mol/dm^3^, the conductivity followed a similar trend by decreasing at a slow rate. However, the conductivity was found to increase because raising DBSA concentration implied increasing the doping level of the PNVC–Ppy copolymer until an optimum concentration of DBSA was reached at 0.015 mol/dm^3^. It then started to decline at a higher doping level, perhaps, as a result of percolation of DBSA molecules into the chain of PNVC–Ppy, which hindered the intermolecular interaction of the copolymer chains that limited the charge carrier mobility across the chains [31]. This same effect, however, facilitates the interaction of PNVC–Ppy with the organic solvent. The solubility of the copolymer, nevertheless, increased with increasing concentration of DBSA because the incorporated surfactant anion (DBSA) expanded the polymer molecule, thereby allowing the organic solvent to diffuse into the spaces between the polymer backbones, and therefore, increased the interaction between the copolymer and the solvent [27]. Besides, PNVC–Ppy was synthesized chemically by using different oxidants and various reaction media [15–17,32]. Although PNVC homopolymer is soluble in a number of organic solvents, its solvating effect did not have any effect on the solubility of PNVC–Ppy prepared earlier [17]. None of the investigations reported that the copolymer had been soluble in any organic solvent. This means that the polypyrrole part of the copolymer had a stronger influence on the overall solubility of the copolymer. Thus, the interaction between Ppy molecule and the molecule of DBSA dopant influenced the solubility of DBSA-doped PNVC–Ppy.

**Figure 6.**
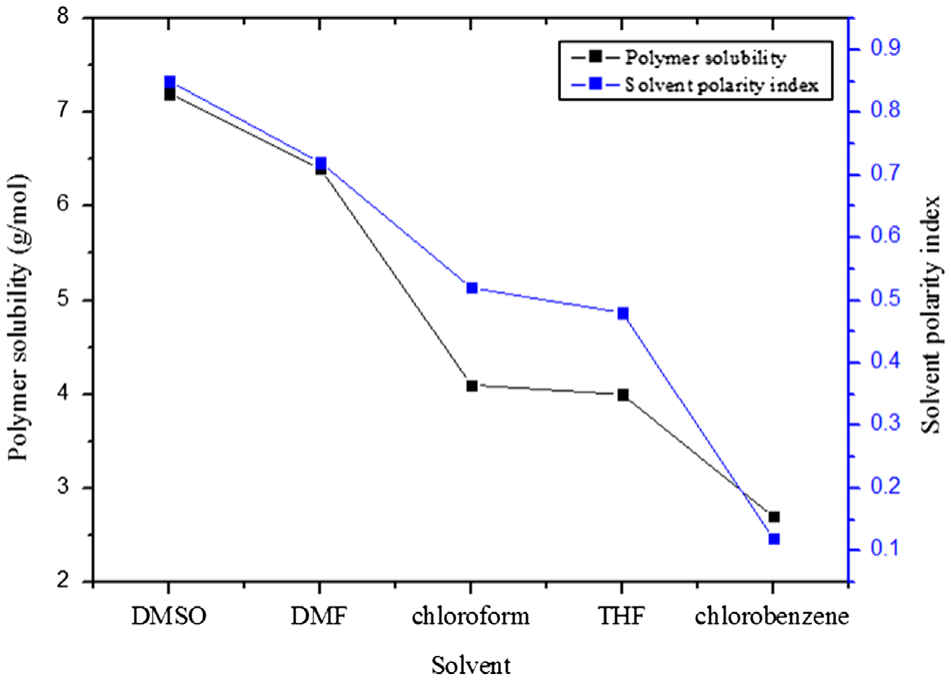
Solubility of DBSA-doped PNVC*–*Ppy vs. polarity indices of the solvents.

Relative solubility of the copolymer in selected organic solvents was further investigated. Table 1 shows the solubility of PNVC–Ppy in the selected solvents. Solubilities with a value <0.6 g/100 ml were considered as partially soluble (PS) and the values within the range of 0.6–0.9 g/100 ml were termed soluble (S). It should be noted that despite the presence of long alkyl chain of DBSA, which induced polymer-solvent interaction, the copolymer dissolved in the selected solvents based on the polarity indices of the respective solvents (Figure 6).

**Table 1.** Solubility of PNVC*–*Ppy in selected organic solvents (g/100 ml).

Solvent	DMSO	DMF	Chloroform	THF	Chlorobenzene
PNVC*–*Ppy	0.85	0.72	0.52	0.48	0.12
Polarity index	7.2	6.4	4.1	4.0	2.7

Notes: IS = insoluble; PS = partially soluble; S = soluble.

The dissolution of the copolymer in high polar solvents, such as DMSO and DMF, was facilitated by hydrogen bond interaction between the DBSA of the copolymer and the solvent. Thus, no such strong hydrogen bond interaction was detected between the copolymer and the weakly polar solvents (PI = 4.1) or relatively non-polar solvent (PI = 2.7).

### 3.3. Optical properties of DBSA-doped PNVC–Ppy

**Figure 7.**
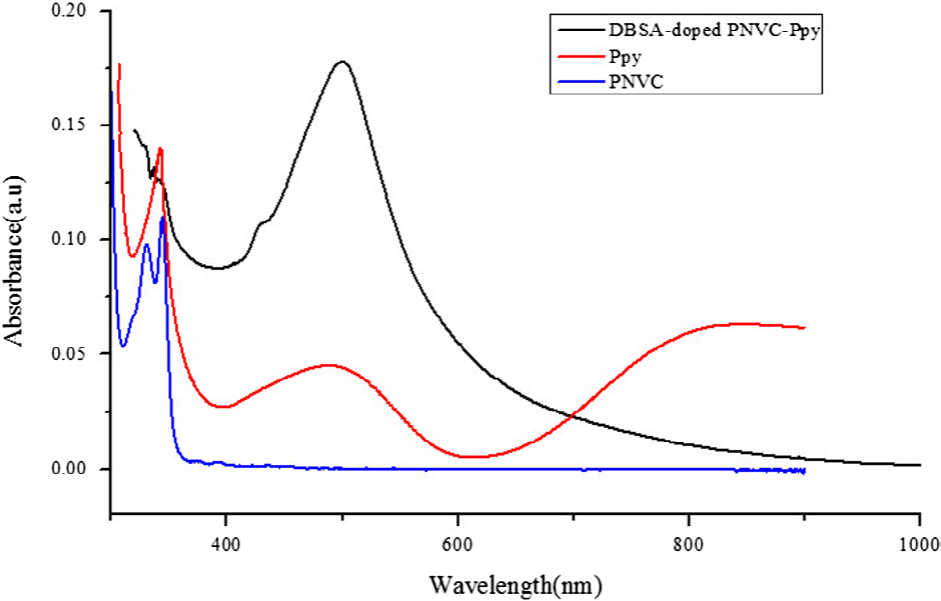
Optical absorption spectra of PNVC, DBSA-doped PNVC–Ppy, and Ppy.

**Figure 8.**
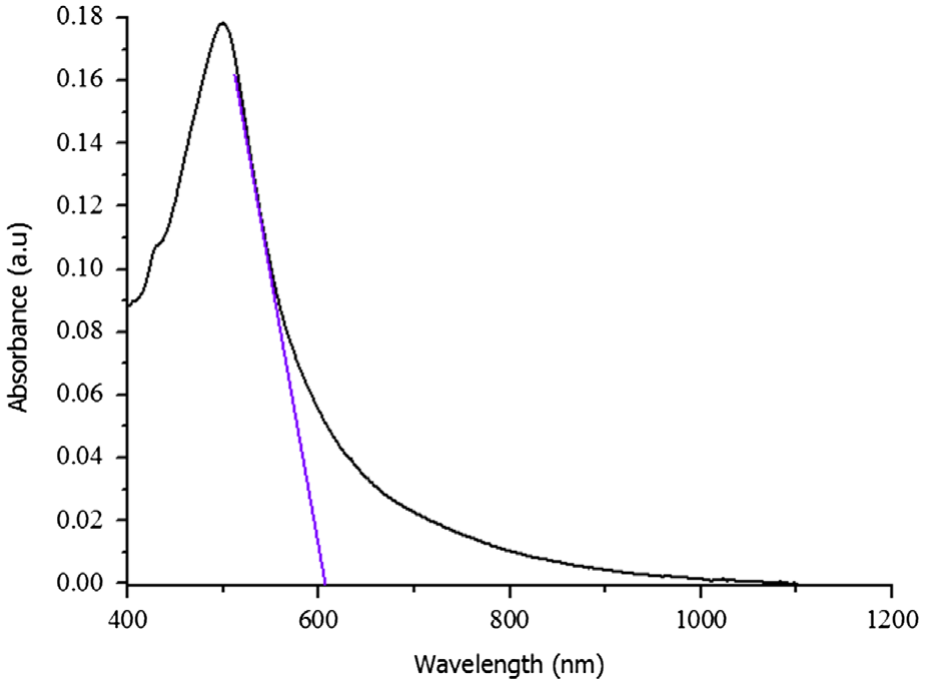
Band gap estimation of DBSA-doped PNVC*–*Ppy.

The UV–vis spectra recorded for doped PNVC–Ppy, PNVC, and Ppy samples in chloroform are illustrated in Figure 7. The solution of Ppy in chloroform (Figure 7(b)) projected a strong absorption peak at 340 nm, one spectra band of lower intensity between 436 and 645 nm, and a free carrier tail at 907 nm. The peak at 340 nm was attributed to the excitation of *π*–π* transition in the pyrrole ring. Besides, the broad peak at 480 nm corresponded to the pyrrole polaronic band, while the broad free carrier tail was due to bipolaronic transition. Furthermore, the presence of polaron and bipolaron in the spectra of polypyrrole confirmed that the polypyrrole had been doped with DBSA. Polaron and bipolaron are known to be charge carriers in doped Ppy. Therefore, high conductivity of doped polypyrrole was due to band gap reduction because of formation of (bi)polaron [33,34]. Moreover, typical absorption spectra of PNVC were observed at 295, 328, and 345 nm in the UV region of electromagnetic spectrum (Figure 7(c)) [13,35]. The free carrier tail, which is responsible for high degree of conjugation in doped polypyrrole, was not found in the spectra of DBSA-doped PNVC–Ppy (Figure 7(a)). The presence of PNVC in the polymer chain of polypyrrole shortened the length of the polypyrrole conjugation, and therefore, the conductivity of the DBSA-doped PNVC–Ppy was reduced relative to that of polypyrrole because the incorporation of NVC into the chain of pyrrole affected the mobility of charge carrier along the Ppy backbone. This observation is in good agreement with the result obtained from the electrical conductivity test of the copolymer. Meanwhile, the optical band gap of the copolymer was estimated to be 2.04 eV as deduced using the absorption spectrum fitting method (Figure 8). Compare with band gap of Ppy (~2.7 eV) and PNVC (3.6 eV), the new copolymer exhibits a reduced band gap, indicating that PVNC truly incorporated in the Ppy chain to form the new copolymer. To select a polymer as a potential active layer material for optoelectronic devices, solution processability and moderate band gap are parts of the criteria. Thus, the DBSA-doped PNVC–Ppy under study can be applied for the purpose.

**Figure 9.**
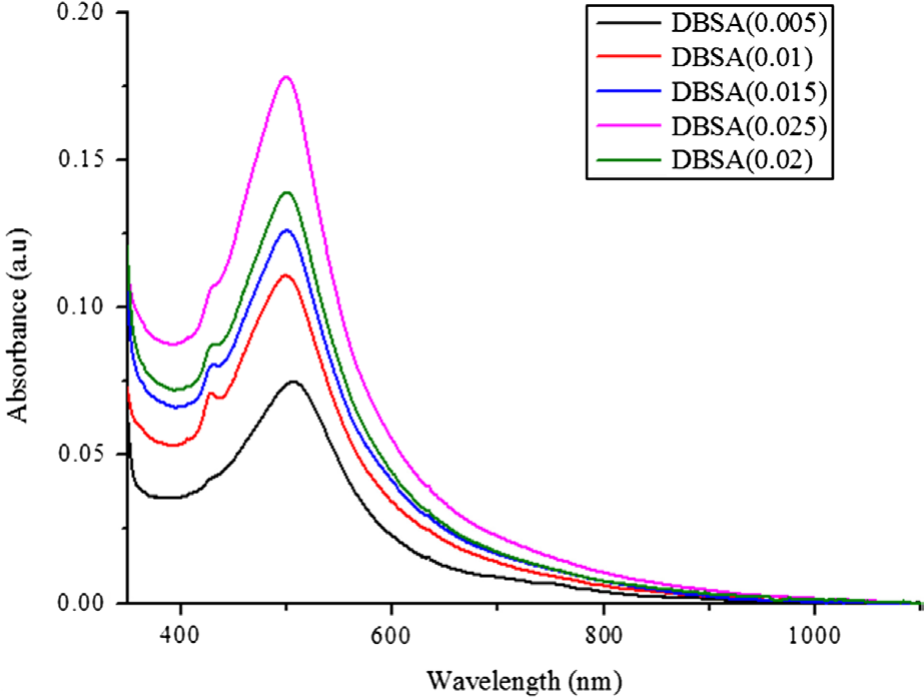
UV–vis spectra of BDSA doped PNVC*–*Ppy at varying concentrations of DBSA.

Figure 9 shows the effects of varying DBSA concentrations on the UV–vis absorption of DBSA-doped PNVC*–*Ppy. Maximum absorption of all the spectra appeared at relatively similar wavelength (500 nm), but with different levels of intensities. The intensities varied with the concentration of the dopant and the copolymer doped with the highest concentration of DBSA recorded the highest conductivity.

### 3.4. Infrared analysis of DBSA-doped PNVC–Ppy

**Figure 10.**
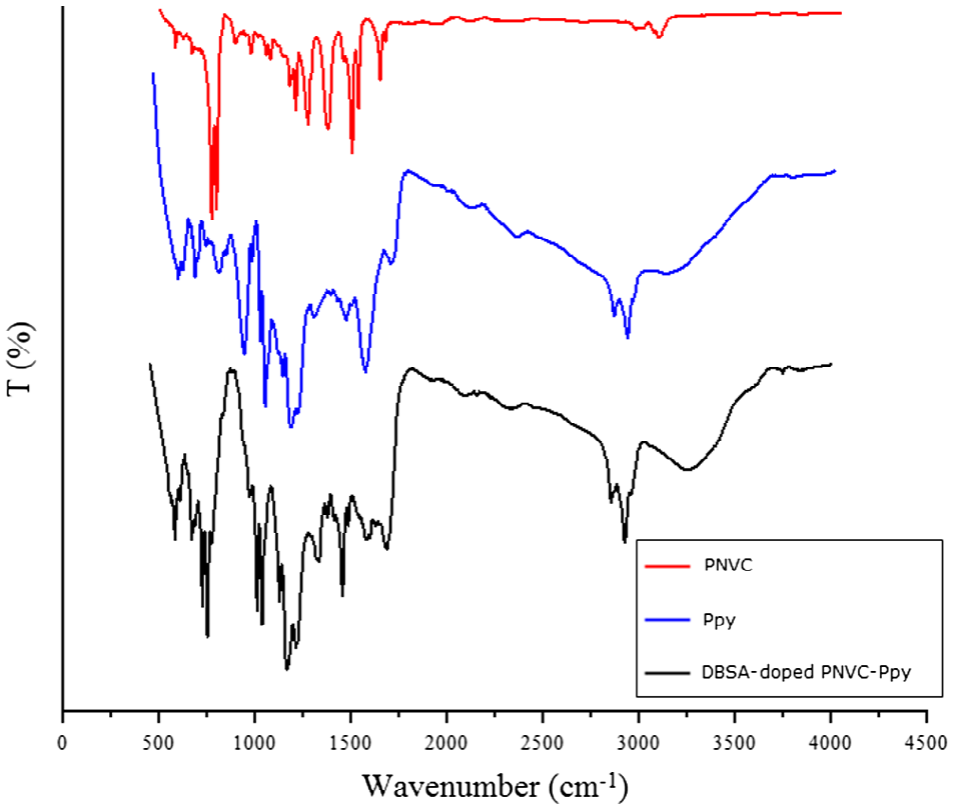
FTIR spectrum of PNVC, Ppy, and DBSA-doped PNVC*–*Ppy.

The FTIR spectra of PNVC, Ppy, and DBSA-doped PNVC*–*Ppy are shown in Figure 10, while the details of the assigned peaks are presented in Table 2. The band at 1454 cm^−1^ was attributed to C=C stretching vibration of polypyrrole ring. Also, the bands at 770 and 668 cm^−1^ corresponded to C–C out of plane ring deformation or C–H rocking of Ppy. In addition, the band located at 1162 cm^−1^ was assigned to C–N stretching vibration of Ppy [9]. The next band at 1330 cm^−1^ was ascribed to C–C stretching vibration of Ppy moiety. The absorption at 2918 cm^−1^ was attributed to C–H asymmetric vibration. Hence, the presence of these bands in the spectra of DBSA-doped PNVC*–*Ppy confirmed the incorporation of PNVC into the backbone of Ppy. Furthermore, the Ppy bands at 1555 and 921 cm^−1^, as well as the PNVC bands at 1596 and 3046 cm^−1^, were not located in the spectra of DBSA-doped PNVC*–*Ppy. In the case of PNVC, the band located at 723 cm^−1^ was attributed to ring deformation of substituted aromatic structure [16,19], whereas the band at 1483 cm^−1^ had been ascribed to ring vibration of NVC moiety. Generally, the peak intensities of copolymer were lowered compared to PNVC. This indicated that not only pyrrole participated in the oxidation process, but carbazole also oxidized via carbazole ring upon the addition of APS oxidant to form the copolymer. Moreover, bands at 1035 and 1008 cm^−1^ were observed in the spectrum of the copolymer, were due to S=O and benzoid ring of DBSA. These bands confirmed that the PNVC*–*Ppy was in doped state [36].

**Table 2.** FTIR band assignments of PNVC, PPY, and PNVC–Ppy.

Ppy	PNVC*–*Ppy	PNVC	Peak assignment
–	–	3046	C–H asymmetric stretching of aromatic structure
2916	2918		
2848	2851		
–	1664	1626	C=C stretching vinylidene group
–	–	1596	
1555	–	–	C=C stretching of polypyrrole ring
1454	1454	1452	
1165	1162	1155	C–H in plane deformation of aromatic ring
1123	1125	1125	
790	770	–	C–C out of plane ring deformation or C–H rocking
668	668	–	
1287	1330	1323	=C–N planar vibration
1032	1035	1025	S=O of DBSA
1009	1008	1004	Benzoid ring of DBSA
–	1483	1482	Ring vibration of NVC moiety
–	749	743	>CH_2_ rocking vibration due to tail to tail addition
–	723	718	Ring deformation of substituted aromatic structure

### 3.5. XRD pattern of DBSA-doped PNVC–Ppy

**Figure 11.**
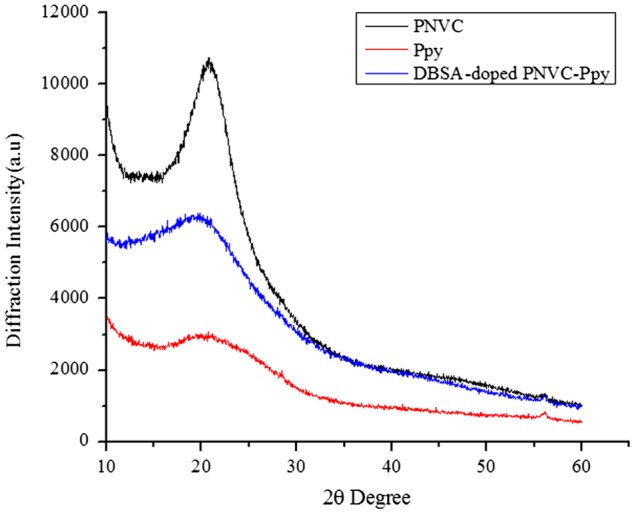
XRD patterns of PNVC, Ppy, and DBSA-doped PNVC*–*Ppy.

XRD measurements were performed to investigate the crystal structure of DBSA-doped PNVC*–*Ppy. The XRD patterns of PNVC, Ppy, and the copolymer are shown in Figure 11. The broad diffraction band of Ppy, which appeared at 2*θ* = 20.1°, clearly indicated that polypyrrole was totally amorphous. The pattern of PNVC was dominated by a narrower and a more intense peak at 2*θ* = 20.8°, which revealed the presence of semi crystalline phase. As for the copolymer, the broad peak centred at 2*θ* = 19.3° revealed that the DBSA-doped PNVC*–*Ppy was amorphous. Moreover, the interaction between the NVC and the monomers, which gave rise to the copolymer, was confirmed by a peak shift of PNVC from 2*θ* = 20.8°–19.3° of the copolymer upon copolymerization.

### 3.6. Thermal property of the DBSA-doped PNVC–Ppy

**Figure 12.**
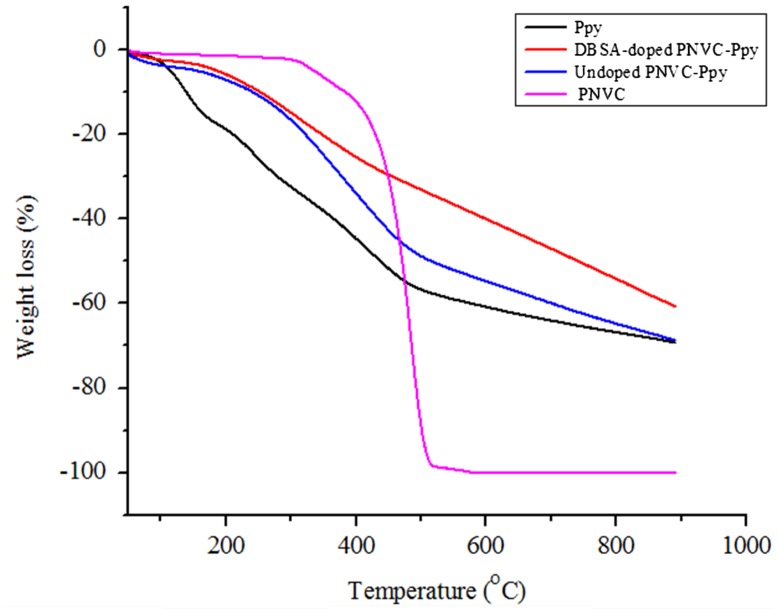
TGA thermogram of PNVC, Ppy, doped PNVC*–*Ppy, and undoped PNVC*–*Ppy.

The thermal behavior of DBSA-doped PNVC–Ppy was investigated via TGA analysis under nitrogen atmosphere. Figure 12 shows the TGA curves for the homopolymers, as well as the DBSA-doped and undoped copolymers. The measurement was taken at a temperature range of 50–900 °C at 20.00 °C/min. Ppy was the least stable to heating with the highest rate of decomposition. The first stage of decomposition started at 81 °C and it lost 16% of its weight at 173 °C. Decomposition at this stage is usually attributed to loss of physical moisture content [37]. Further decomposition between 173 and 500 °C, where Ppy has lost almost 60%, was due to degradation of polypyrrole backbone and other interchain bonds. Between 500 and 900 °C, Ppy lost 70% of its total mass, leaving behind carbonized polymer residue, a phenomenon that is common to infusible conjugated polymers [38].

Conjugated polymers possess high carbon content due to double bonds alternation. Hence, thermal stability of undoped PNVC*–*Ppy was enhanced. Besides, the effect of the DBSA dopant on thermal stability of the PNVC*–*Ppy was revealed by the thermogram of DBSA-doped PNVC–Ppy. Although, both doped and undoped PNVC–Ppy had initial thermal degradation at the same temperature range (50–200 °C), DBSA-doped PNVC–Ppy appeared to be more thermally stable because 75% of its weight remained after the second stage of thermal degradation, which occurred between 200 and 400 °C. Other than that, the decomposition of polymer backbone took a longer temperature range (200–470 °C) in undoped PNVC–Ppy and almost half the polymer weight had already lost to thermal degradation. Moreover, superior thermal stability of PNVC carbazole was displayed as it remained stable until up to 300 °C with less than 2% loss of surface moisture content. However, PNVC decomposed completely before 600 °C with no carbonized residue after decomposition, as indicated by the thermogram. In summary, not only PNVC contributed to thermal stability of DBSA-doped PNVC–Ppy, but presence of DBSA in the copolymer molecule also further enhanced the thermal stability of the copolymer.

### 3.7. Morphology of DBSA-doped PNVC–Ppy

**Figure 13.**
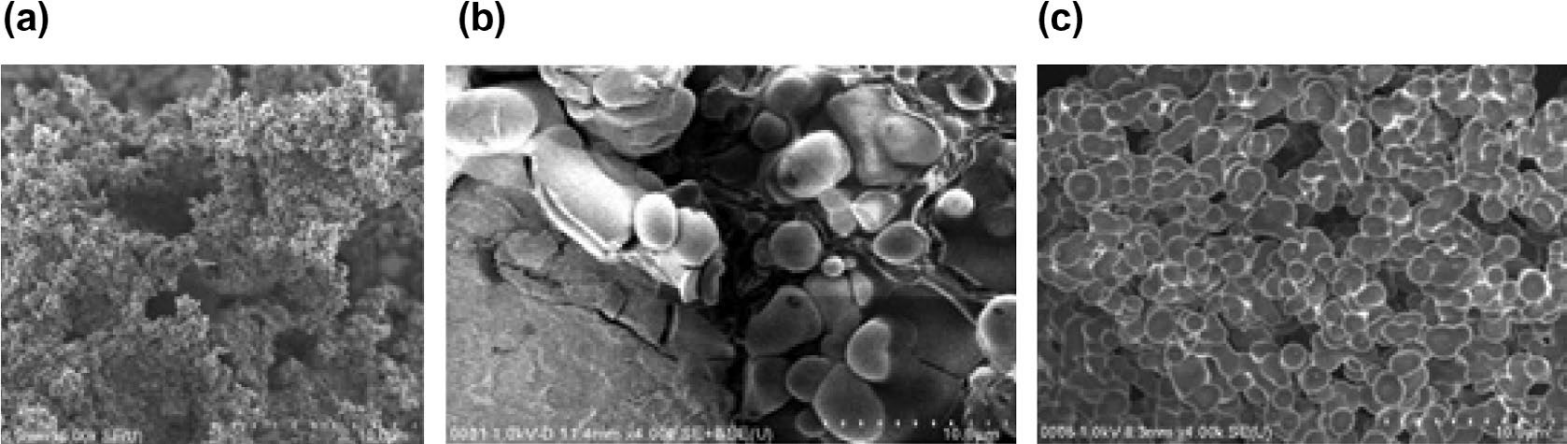
FESEM images of (a) Ppy, (b) PNVC, and (c) DBSA-doped PNVC–Ppy copolymer.

Figure 13 shows the SEM images of PNVC, Ppy, and DBSA-doped PNVC*–*Ppy all at 10 μm magnification. PNVC showed spherical particles of larger size. The morphology revealed less tendency for agglomeration, which is perhaps the reason why PNVC was readily soluble in common organic solvents. Besides, preparation condition and method usually influence the surface morphology of Ppy [34]. Ppy also displayed a porous morphology with agglomeration of tiny globules. Meanwhile, the DBSA-doped PNVC*–*Ppy presented a morphology that completely differed from those of homopolymers. PNVC*–*Ppy copolymer showed a distinctly formed and densely packed globular structure without voids in-between. The particles were uniformly arranged with similar diameters. The uniform size revealed that copolymerization actually took place between NVC and pyrrole monomers; forming a new product with distinctively a different structure.

## 4. Conclusion

In summary, this study had demonstrated that intractable copolymer of NVC and pyrrole could be rendered soluble in polar organic solvents by the counter ion effect of DBSA. DBSA-doped PNVC*–*Ppy can now be seen as a solution processable conducting polymer, which can be simply obtained via chemical oxidation of NVC and Py monomers using APS oxidant. The new soluble copolymer should be better than PNVC in terms of electrical conductivity and optical absorption; while Ppy in terms of thermal stability. Furthermore, the solubility of DBSA-doped PNVC*–*Ppy in some polar organic solvents allows further studies to look into its interaction with other organic solvents that are less polar in order to widen its area of applications. In fact, the easy synthetic route, solution processability, and low band gap of the soluble copolymer further enable the study of film forming ability of this product for applications as an active layer material or transparent electrodes in optoelectronics.

## Disclosure statement

No potential conflict of interest was reported by the authors.

## Funding

This work was supported by the University of Malaya Postgraduate Research Fund (PPP) [grant number PG064-2013A] and the Ministry of Higher Education, Malaysia for providing Fundamental Research Grant Scheme (FRGS) [project number FP034-2013A].
